# Detection, treatment, and course of eating disorders in Finland: A population-based study of adolescent and young adult females and males

**DOI:** 10.1002/erv.2838

**Published:** 2021-05-18

**Authors:** Yasmina Silén, Pyry N. Sipilä, Anu Raevuori, Linda Mustelin, Mauri Marttunen, Jaakko Kaprio, Anna Keski-Rahkonen

**Affiliations:** 1 Clinicum, Department of Public Health, University of Helsinki, Helsinki, Finland; 2 Department of Adolescent Psychiatry, University of Helsinki and Helsinki University Central Hospital, Helsinki, Finland; 3 Institute for Molecular Medicine Finland (FIMM), University of Helsinki, Helsinki, Finland

**Keywords:** course, DSM-5, detection, eating disorders, treatment

## Abstract

**Objective:**

We assessed the detection, treatment and outcomes of DSM-5 eating disorders in a nationwide community setting.

**Method:**

The FinnTwin12 cohort comprises twins born in 1983–1987 in Finland (*n* = 5,600), with follow-up starting at age 12. We outline treatment and outcomes of the 127 females and 15 males diagnosed with a lifetime DSM-5 eating disorder in interviews conducted for a subsample (*n* = 1,347) in their early 20s.

**Results:**

Only 45 (32%) of those diagnosed with eating disorder in the interviews had their condition detected in healthcare, and even fewer received treatment (30% of females, 13% of males). Anorexia nervosa (AN), bulimia nervosa, and atypical AN were detected and treated more often than other eating disorders. Five years after disease onset, 41% of those diagnosed had recovered. There were no statistically significant differences in the course of different eating disorders (log-rank *p* = 0.66) but the outcome was more favourable among males (log-rank *p* = 0.008). The likelihood of 5-year recovery did not differ between those who had and who had not received treatment (41.1% vs. 40.5%, log-rank *p* = 0.66).

**Conclusion:**

Although eating disorders are common and symptoms are persistent for many, they remain under-diagnosed and under-treated. In real-world settings, effectiveness of provided treatments may be limited.

## 1 | INTRODUCTION

Eating disorders are common in the community. The lifetime prevalence estimates of interview-based community studies have ranged from 3.1% to 17.9% among females and 0.6% to 2.4% among males in the DSM-5 era (Fairweather-Schmidt & Wade, 2014; Micali et al., 2017; Mustelin, Lehtokari et al., 2016; Mustelin, Silen, et al., 2016; Silén et al., 2020; Smink et al., 2014; Stice et al., 2013; Udo & Grilo, 2018; Wade & O’Shea, 2015; Wagner et al., 2017). Still, only a minority of individuals with eating disorders have their conditions detected and receive appropriate mental healthcare (Hart et al., 2011; Treasure et al., 2020). Moreover, the perceived need for help, detection and treatment of eating disorders has been guided by stereotypes of young, skinny women, although eating disorders affect individuals of all ages, genders and body sizes (Mangweth-Matzek & Hoek, 2017; Mulders-Jones et al., 2017; Schaumberg et al., 2017; Sonneville & Lipson, 2018).

A major shortcoming in the current knowledge is that most studies of the detection, treatment and course of eating disorders have focused on females, leading to gender bias (Bardone-Cone et al., 2018; Murray et al., 2017). In addition, research has been centred on anorexia nervosa (AN) and has seldom included estimates for bulimia nervosa (BN), binge eating disorder (BED), and other specified or unspecified feeding or eating disorders (OSFED, UFED; Bardone-Cone et al., 2018; Keel, 2018; Smink et al., 2013). Furthermore, the natural course of eating disorders, still, remains poorly understood (Brown et al., 2015). Lastly, studies investigating the association between interventions and eating disorder outcomes are limited and focus mostly on clinical samples.

To address these knowledge gaps, we assessed how DSM-5 classified eating disorders were detected and treated by healthcare providers among males and females in the community. Our aim was also to estimate the likelihood of recovery in various diagnostic subgroups of eating disorders and among treated versus untreated participants. We used data from the FinnTwin12 birth cohort, a longitudinal nationwide community-based study. In their early twenties, more than 1,300 males and females were interviewed using a standard semi-structured diagnostic interview.

## 2 | METHODS

### 2.1 | The FinnTwin12 birth cohort and intensively studied sample

The FinnTwin12 study is a longitudinal population-based study that comprises Finnish twins born in 1983–1987 (*n* = 5600, identified from the Finnish Central Population Registry). The twins were approached at ages 12, 14, 17.5 and 22 years. In all waves, the response rate was 85%–90% (Rose, Salvatore, et al., 2019).

The study includes an epidemiological sample (all participants) and an intensively studied sample (selected from the epidemiological sample). In the intensively studied sample (1,035 families), most families were selected randomly (72.3%, 748 families), and the rest (27.7%, 287 families) were enriched with families where parents showed signs for possible alcohol problems based on elevated scores on the Malmö-modified Michigan Alcoholism Screening Test (Mm-MAST; Seppä et al., 1990). The enrichment and other aspects of the study protocol have been described previously in more detail (Rose et al., 2004; Silén et al., 2020).

Data collection and analysis were carried out in accordance with the Declaration of Helsinki. The ethics committee of The Hospital District of Helsinki and Uusimaa (HUS) and the Institutional Review Board of Indiana University approved the study protocol, and all participants gave written informed consent.

### 2.2 | DSM-5 eating disorder diagnoses

During the fourth wave at the mean age of 22.4 years (2006–2009), the intensively studied subsample of twins (*n* = 1,347, including 709 females and 638 males, 73% of the target sample of twins interviewed at age 14) were interviewed using the eating disorder section of the Structured Clinical Interview for DSM-IV (SCID; First et al., 2002). All interviewers were healthcare professionals (registered nurses, graduate students in psychology, or masters of healthcare); the majority of the interviews were organized face-to-face (*n* = 709), the rest by telephone. The interviewers had written a detailed description of each participantś symptoms in their case notes; three experienced medical doctors (YS, AR, AK-R) reviewed interview scores and case notes and made consensus DSM-5 diagnoses (American Psychiatric Association, 2013). In total, 127 females and 15 males were diagnosed with a lifetime DSM-5 eating disorder (Silén et al., 2020). This amounted to a lifetime prevalence of 17.9% (one in six) for females and 2.4% (one in 40) for males (Silén et al., 2020). Three individuals had both AN and BN diagnoses. In 15 twin pairs (nine monozygotic) comprising 30 individual twins, both twins were diagnosed with an eating disorder. Our sensitivity analyses showed no statistically significant difference between the lifetime prevalence of eating disorders in the enriched and randomly selected samples among females (15.6% vs. 18.7%, *p* = 0.37) or among males (3.8% vs. 1.8%, *p* = 0.16; Silén et al., 2020).

### 2.3 | Detection in healthcare and treatment received

During the interview, participants were asked if any healthcare professional had ever diagnosed them with an eating disorder. Furthermore, they were asked about any eating disorder treatment that they had received. The treatment was categorized into partially overlapping groups: hospital treatment, outpatient treatment, medication, individual psychotherapy or family therapy, and treatment at a specialized eating disorder unit. Outpatient treatment was defined as any treatment appointments related to an eating disorder other than treatment in psychiatric or somatic hospital wards.

### 2.4 | Full recovery from eating disorder symptoms

At the end of the eating disorder assessment, the participants were asked about the time course of their symptoms. Participants were asked at what age their eating disorder symptoms had started, whether they were still suffering from eating disorder symptoms, and if not when they thought they had recovered. They were also asked what had helped them to recover. Interviewers wrote a detailed description in their case notes.

The criterion for recovery was multidimensional. To be classified as recovered, participants themselves had to express that they thought they no longer suffered from an eating disorder. If a participant reported that he or she had still been suffering from any eating disorder behaviour (e.g., restrictive eating or binge eating, compensatory behaviours, excessive exercise) or psychological symptoms (e.g., persistent body image concerns, fear of weight gain, fatphobia) during the last year, they were considered still having symptoms (not recovered). Since eating disorders sometimes involve denying the seriousness of symptoms, for a participant to be classified as recovered, participants’ description of recovery had to be clinically meaningful; moreover, a current body mass index (BMI) of 18.5 kg/m^2^ or higher was required.

### 2.5 | Statistical analysis

We conducted Pearson chi-squared tests for cross-tabulations and adjusted the *p*-values for the sampling of twins within twin pairs. Recovery rates were analysed using Kaplan-Meier analysis and life tables, and differences in the likelihood of recovery between subgroups were assessed using log-rank tests. We also used Cox proportional hazards models to calculate hazard ratios when analysing the role of eating disorder subtypes in the recovery estimates between sexes. We used Schoenfeld residuals to test that the proportional hazards assumption was not violated. Because of missing data on the onset of an eating disorder for two participants, recovery analysis included 140 participants. All analyses were conducted using Stata Statistical Software, version 14.

## 3 | RESULTS

### 3.1 | Eating disorder detection in health-care

There were 142 community-based participants diagnosed with a lifetime DSM-5 eating disorder in our interviews. For the 140 participants for whom we had data on eating disorder onset, the mean age of onset was 16.5 years (standard deviation [SD] 2.9, range 9–22). They were followed up for recovery for a mean of 4.0 years (SD 2.9, range 0.5–13). Of the 142 participants with an eating disorder, 45 (32%) had had their disorder detected by healthcare professionals. The likelihood of detection was similar for females (41 [32%]) and males (4 [27%]), chi-square *p* for difference 0.7. AN (57%), BN (50%), and OSFED atypical AN (44%) were detected more often than were BED (33%), other OSFEDs (12%), or UFED (5%) (Table 1), chi-square *p* for difference 0.0001.

### 3.2 | Received treatment for eating disorder

Forty participants received at least some treatment for an eating disorder (89% of those detected by healthcare professionals, 28% of those diagnosed in the present study). In detail, 30% of females received treatment (*n* = 38) versus 13% of males (*n* = 2), chi-square *p* for difference 0.19. The likelihood of being detected by healthcare professional but not receiving treatment was more marked among males among males (*n* = 2 [50%]) than among females (*n* = 3 [7%]); chi-square *p* for difference 0.03. Treatment was more common in AN (52%), BN (50%), and OSFED AN (38%) than in BED (17%), other OSFEDs (12%), or UFED (2%; Table 1), chi-square *p* for difference 0.0001.

All treated participants had received outpatient treatment. Only 5.6% (*n* = 8) of those diagnosed with an eating disorder had received inpatient treatment (Table 1). Of them, six had been diagnosed with AN only, one with both AN and BN, and one with BN only.

Twelve individuals (8.5% of those diagnosed) reported receiving psychotropic medication for the treatment of an eating disorder (Table 1). The medications received were the following: fluoxetine (7), sertraline (2), citalopram (1), mirtazapine (1), and quetiapine (2). The medication was most commonly associated with AN and BN as only one out of the 81 participants with an eating disorder outside these diagnostic groups had received medication for the treatment of his or her eating disorder.

Nine participants (7% of those diagnosed) had received individual psychotherapy, and one family therapy (Table 1). Psychotherapy was most common with participants suffering from AN (7/46 [15%]).

Six participants (4% of those diagnosed) had received treatment at a unit that was specialized in the treatment of eating disorders (Table 1). A total of 19 participants (13% of those diagnosed) reported that a school nurse had been involved in the detection or treatment of their eating disorder. In total, school nurses were involved in 42% of the cases detected in healthcare.

### 3.3 | COURSE

#### 3.3.1 | Eating disorder symptoms at the time of the interview

Of those with a lifetime DSM-5 eating disorder, 84/127 females (66%) and 4/15 males (27%) had suffered from eating disorder symptoms during the last 12 months preceding the interview in their early 20s.

#### 3.3.2 | The course of eating disorders, 5-year recovery rate and mean duration

**FIGURE 1:**
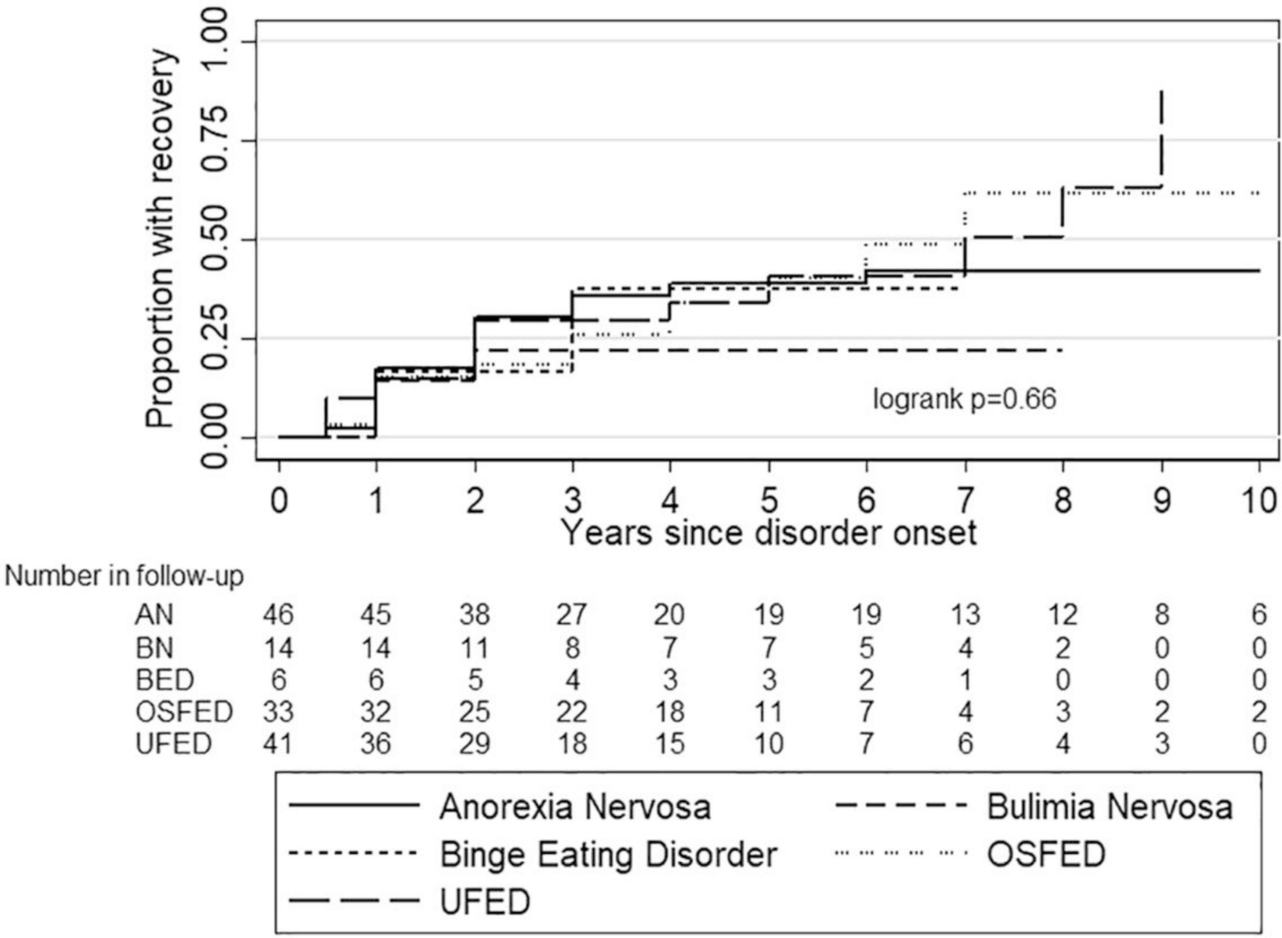
Recovery from anorexia nervosa (AN), bulimia nervosa (BN), binge eating disorder (BED), other specified feeding and eating disorders (OSFED) and unspecified feeding and eating disorders (UFED). Females and males were analysed together

Figure 1 shows the course of each eating disorder for both genders combined. Both genders were analysed together because of the small number of males. There were no statistically significant differences in the course of different eating disorders (log-rank *p* = 0.66) or OSFED subgroups (log-rank *p* = 0.60, Figure S1). Table 2 shows the 5-year recovery rates for each diagnosis for both genders combined and the mean duration of each specific eating disorder at the time of the interview among those who were fully recovered and for the whole sample.

**FIGURE 2:**
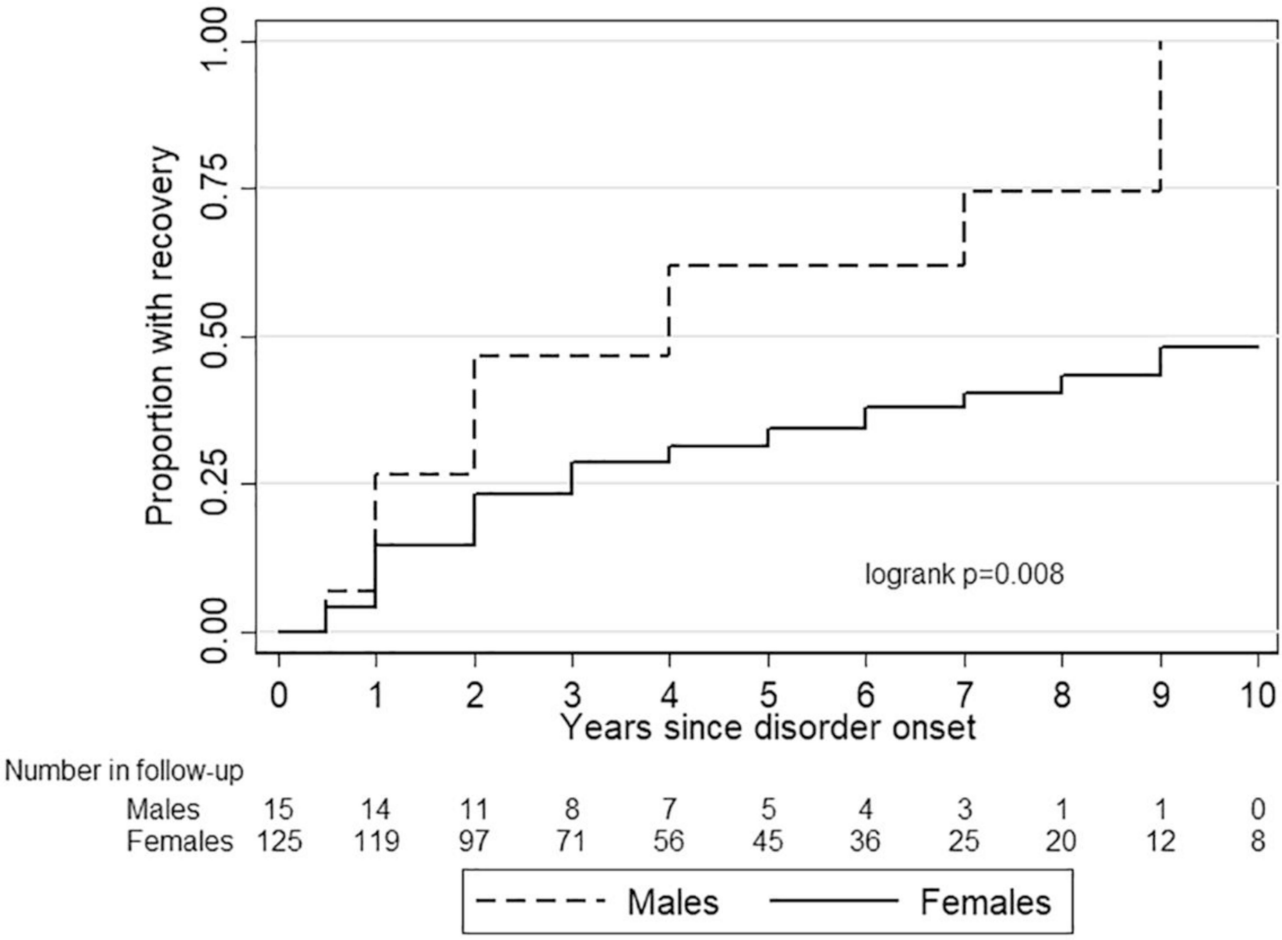
Recovery from DSM-5 eating disorders among females versus males

The 5-year recovery rate of all eating disorders combined was 40.7%; 64% among males, and 37% among females. In general, males had a shorter duration of eating disorder symptoms and recovered more often than did females (log rank *p* = 0.008; Figure 2). This difference in recovery was not explained by the different distribution of eating disorder subtypes between males and females. The hazard ratio for recovery was 2.3 (95% Cl 1.3–4.0) for males versus females in unadjusted models and 2.2 (95% Cl 1.1–4.2) in models adjusted for ED subtypes.

#### 3.3.3 | Association between treatment and course of eating disorders

**FIGURE 3:**
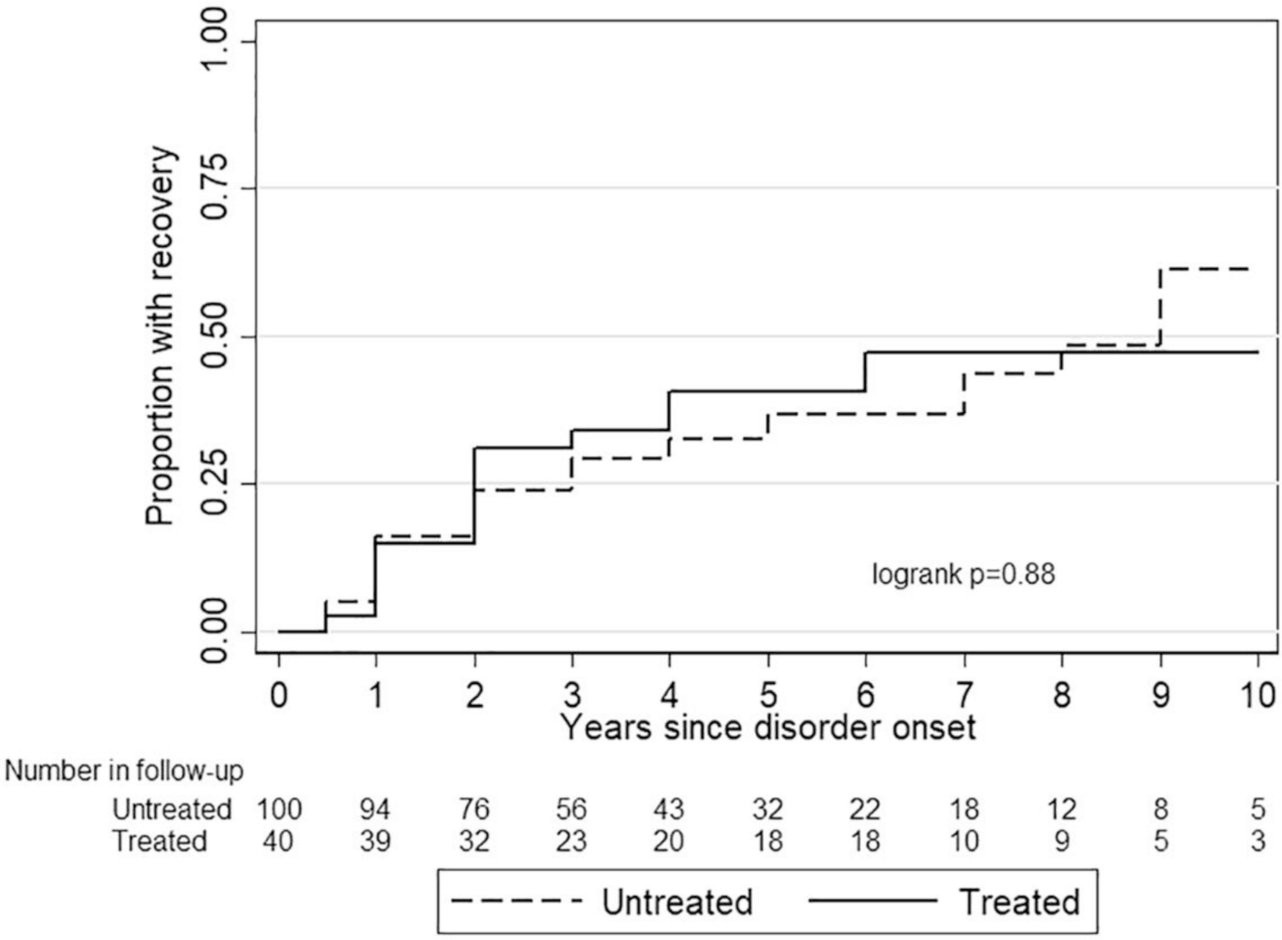
Recovery from eating disorders by those who received treatment for eating disorder versus those who remained untreated. Females and males were analysed together

Figure 3 shows the course of eating disorders in those who received treatment for their eating disorder versus those who remained untreated. The Figure S2 shows the course for each eating disorder. Table 2 shows the 5-year recovery rates in the same groups. Altogether, there was no difference between treated and untreated groups.

Among those with any eating disorder, the 5-year recovery rate was 41.1% for the treated versus 40.5% for the untreated (log rank *p* = 0.66).

## 4 | DISCUSSION

Our results show that while DSM-5 eating disorders affect one in six females and one in 40 males in the community, only one-third of those affected are detected by healthcare providers, and even fewer receive treatment. Detection and treatment disparities are evident as atypical disorders are rarely recognized and treated even more rarely. Furthermore, many individuals with an eating disorder seem to suffer from symptoms for years. Five years after disease onset, less than two-fifths of females and two-thirds of males had recovered. We did not observe significant differences in the course of different diagnostic groups. Additionally, the likelihood of a 5-year recovery was around 40 percent in both treated and untreated groups. Together, our results indicate that the commonness and persistence of eating disorders are not matched with healthcare efforts.

### 4.1 | The detection of DSM-5 eating disorders by the healthcare system

Supporting previous observations, we found that most adolescents and young adults who meet the diagnostic criteria for eating disorders are not detected by the healthcare system (Hart et al., 2011; Micali, N. et al., 2017; Mohler-Kuo et al., 2016; Mustelin, Lehtokari et al., 2016; Mustelin, Silen, et al., 2016; Solmi et al., 2016; Smink et al., 2014). The likelihood of detection was similar among females and males—between a quarter and one-third of cases were detected. Furthermore, in line with previous studies (Mustelin, Lehtokari et al., 2016; Mustelin, Silen, et al., 2016; Smink et al., 2014), eating disorders with typical representations, such as AN, BN and atypical AN, were more often detected and subsequently treated than other specified or unspecified eating disorders.

The poor detection rate of eating disorders is a multidimensional issue. Many individuals with eating disorders do not recognize the severity of their symptoms and the need for treatment, or fear that their symptoms will be belittled (Cachelin & Striegel-Moore, 2006; Grillot & Keel, 2018). Identifying an eating disorder and referring a patient for treatment can also be difficult for healthcare professionals due to a shortage of available treatments and a lack of skills in both primary and secondary healthcare (Waller et al., 2014). Increasing detection of eating disorders requires public discussion to reduce the stigma associated with these disorders and education of both the public and healthcare professionals about the diversity of symptoms and weights, treatment options, and available specialized outpatient facilities (Duncan et al., 2017; House et al., 2012; Sangha et al., 2019; Waller et al., 2014).

### 4.2 | Actualized treatment

In our sample, only one out of four had received any treatment for their eating disorder. The scarcity of treatment in our setting matches previous reports, indicating a significant discrepancy between the need for help and the available services (Micali et al., 2017; Solmi et al., 2016; Smink et al., 2014; Wagner et al., 2017). Previous literature indicates that males seek and receive treatment less often for eating disorders than females (Limbers et al., 2018; Mangweth-Matzek & Hoek, 2017; Sonneville & Lipson, 2018). In our sample, the change of being detected in healthcare but not receiving treatment was more marked among males. However, the difference in the actual treatment rates was not statistically significant.

In Finland, schools at all levels, including universities, have health and welfare services to support the well-being of all students. In our sample, 42% of those detected by healthcare providers reported that a public health nurse working in school health had been involved in the detection or taken part in the treatment. The true rate is probably even higher because none of the participants who had received eating disorder treatment at a specialized unit or at the hospital ward mentioned school nurses’ appointments, although many referrals likely go through the school health system to tertiary care. In any event, school nurses seem to have an important role in the detection and treatment of eating disorders in Finland, although their roles may not exist or be the same in other countries. Raising the awareness of the commonness and diversity of eating disorder symptoms among those healthcare professionals who work closely with adolescents and young adults could be an effective way to improve the detection and treatment rates of eating disorders.

### 4.3 | The natural course of DSM-5 eating disorders

We found that after 5 years after eating disorder onset, approximately 40% of those diagnosed were recovered in each diagnostic group except that only 20% of those with BN had recovered. Although this difference was not statistically significant, the persistence of BN symptoms has been well-documented in some previous studies (Agras et al., 2009; Glazer et al., 2019; Keski-Rahkonen et al., 2009; Kessler et al., 2013). Moreover, the persistence of symptoms in OSFED and UFED emphasizes the seriousness of these diagnoses and supports some previous findings (Mustelin, Lehtokari et al., 2016; Wade & O’Shea, 2015). Adding to the scarce literature to date (Riesco et al., 2018), we did not find significant differences in the outcomes of different OSFED subtypes, although these results were based on very few participants in some OSFED subcategories.

Samples, definitions of recovery, measurements, and follow-up times vary widely between studies conducted in the DSM-5 era challenging comparison (Glazer et al., 2019; Kessler et al., 2013; Mustelin Lehtokari et al., 2016; Mustelin, Silen, et al., 2016; Stice et al., 2013; Udo & Grilo, 2018; Wade & O’Shea, 2015). Some studies have found that recovery from eating disorders takes many years: up to 11.4 for AN, 12.2 for BN, and 15.9 for BED (mean years with episode; Udo & Grilo, 2018). Conversely, others have found eating disorder episode durations to average as little as 8 months for AN, 2.9 for BN, 3.3 for BED, 11.6 for atypical AN, 3.5 for subthreshold BN, 3.0 for subthreshold BED, and 5 for purging disorder (PD). The corresponding 1-year remission rates were 75% for AN, 93% for BED, 71% for atypical AN, 94% for PD, and 100% for BN, subthreshold BN, and BED (Stice et al., 2013). Among US adolescent and young adult females, 63% of those with eating disorder had their symptoms remitted 1–3 years after detection (Glazer et al., 2019). In our other Finnish twin sample (FinnTwin 16), the 5-year recovery rate among women was 72% for AN, 60% for OSFED/UFED, and 55% for BN with weekly symptoms (Keski-Rahkonen et al., 2009; Mustelin Lehtokari et al., 2016; Mustelin, Silen, et al., 2016). In sum, our recovery rates seem to be in the lower end and disease duration in the higher end of previous DSM-5 estimates.

The low recovery rates in our study are probably partly due to limits concerning the follow-up time and our strict criteria for recovery. Traditionally, physical dimensions and eating disorder symptoms have been used as a base for the definition of recovery (Vall & Wade, 2015). However, because psychological, emotional, social, and appearance-related aspects seem to be essential for patients (de Vos et al., 2017; Emanuelli et al., 2012), it has been recommended that the definition of recovery should also comprise such aspects (Bardone-Cone et al., 2018). Our definition of recovery was multidimensional, combining participants’ perceptions, BMI, and clinical judgement.

It is noteworthy that a proportion of the non-recovered might have suffered from residual symptoms and no longer fulfilled the full diagnostic criteria for an eating disorder. Moreover, the absence of eating disorder symptoms is not always necessary for patients to consider themselves recovered. Personal recovery often comprises a more holistic perspective, including finding a purpose and identity outside the eating disorder, self-compassion, empowerment, hope, meaning and supportive relationships (Slof-Op ‘t Landt et al., 2019; Wetzler et al., 2020).

Nevertheless, our results seem to align with recovery documented by rigorous long-term studies. After 20 years of follow-up, only approximately 40% of AN and BN patients who had needed inpatient care had achieved remission (Fichter et al., 2017; Quadflieg & Fichter, 2019). Moreover, after 30 years, one in five of those with adolescent-onset AN still had a chronic eating disorder (Dobrescu et al., 2020). Finally, after 12 years, more than 30% of BED patients who had needed inpatient care still met the diagnostic criteria for an eating disorder (Fichter et al., 2008). Together with these results, our findings indicate that the symptoms are persistent for a substantial proportion of eating disorder sufferers, underscoring the seriousness of eating disorders.

The literature on gender differences in eating disorder outcomes is scarce (Strobel et al., 2018; Strobel et al., 2019; Strober et al., 2006). We found that males had a better eating disorder prognosis than did females. However, based on our data, we do not know whether they have better overall psychiatric prognosis. Numerous studies have shown the commonness of heterotypic continuity, that is, one psychiatric condition evolving to a different psychiatric state in the same individual at a later time (Lahey et al., 2014; Raevuori et al., 2009; Shevlin et al., 2017). Among males, the symptoms of the eating disorders may have evolved to some other disorder, like anxiety, depression, or substance use disorder.

### 4.4 | The impact of treatments

We did not find a difference in the likelihood of recovery between individuals who did or did not receive treatment. Our results indicate that many individuals recover from their eating disorders without professional help, and many others suffer from longstanding symptoms despite receiving treatment. Still, our results do not imply that treatment has no effect. The participants were not randomly assigned to receive treatment, and those receiving treatment may have had more severe forms of the disorder in the first place, increasing the probability of detection and subsequent treatment. Without detection and treatment, their outcome might have been less favourable. This confounding by indication has been well described in previous community-based studies assessing other psychiatric conditions (Spijker et al., 2001; van Beljouw et al., 2010). Moreover, our results cannot tell if the actualized treatment was sufficient. For example, only one in 10 of those who had been detected by healthcare professionals received treatment at a unit that was specializing in eating disorder treatment.

Our finding of the weak association of treatment with eating disorder outcome is in accordance with previous longitudinal studies. Two community-based studies have found negligible associations of detection with the outcome of AN and BN (Keski-Rahkonen et al., 2009; Mustelin, Silen, et al., 2016), and one community-based study found no association of treatment with the outcome of DSM-IV defined AN (Dobrescu et al., 2020). Similarly, treatment was not associated with the 5-year outcome among DSM-IV defined AN, BN, and EDNOS patients who were seeking treatment (Ben-Tovim et al., 2001). Together the results indicate that real-world treatments may have limited effectiveness, and implementation studies are vitally important to improve care.

### 4.5 | Limitations and strengths

Our study has some important limitations that should be considered when interpreting our results. First, some aspects of our sample could complicate the generalization of our results. We studied twins, and multiple births are a known risk factor for AN (Goodman et al., 2014). In our study, a subsample was enriched with participants with a family risk for alcohol problems, but the enrichment did not significantly affect eating disorder occurrence (Silén et al., 2020). Second, the age of our sample should be taken account when interpreting our results. Participants had not yet passed the upper limit of the age of peak incidence of eating disorders. In particular, BN and BED tend to have a later onset than does AN (Micali et al., 2013; Mitchison & Hay, 2014; Striegel-Moore & Franko, 2003). Thus, some participants may have been too young for developing an eating disorder or having a sufficient time after its onset to come to healthcare providers’ attention. Moreover, with a longer follow-up, the mean duration would probably have been longer and the recovery rates higher. Third, the numbers of some eating disorder subtypes were too low to make far-reaching conclusions. Fourth, the assessment of symptoms, detection, treatment, and course was retrospective, sometimes occurring years after the original events; this could lead to recall bias. We also only had information about detection and treatment from the respondents; this could lead to information bias. Fifth, we did not have information about comorbid psychiatric disorders. For this reason, we do not know whether eating disorder symptoms were influenced by other psychiatric conditions. Sixth, we did not have information about the impairment related to eating disorders. We do not know whether the onset of OSFED or UFED posed as much of a threat to physical, psychological, and social health compared with, for example, AN or BN. Last, our results did not reflect the most recent detection and treatment efforts.

However, we believe that these limitations are offset by several strengths. Our population-based study design permitted us to assess eating disorder detection, treatment, and course in a naturalistic setting. Furthermore, our assessment protocol was rigorous, including a structured clinical interview of all participants, and our analyses included both genders and a variety of eating disorders. Finally, our definition of recovery was multifaceted, and it included the participants’ self-assessment of recovery.

## 5 | CONCLUSION

Although eating disorders are common and symptoms are persistent for many, only one in three are detected in healthcare, and even fewer receive any treatment. Furthermore, real-world treatments seem to have limited effectiveness. These results underscore the need for immediate actions to improve the detection and treatment of eating disorders.

## Supplementary Material

Supplementary Figure 2

Supplementary Figure 1

## Figures and Tables

**TABLE 1:** Detection and treatment of DSM-5 eating disorders in a population sample of adolescents and young adults

Eating disorder diagnosis (*N*)	Gender	Participants diagnosed in the present study	Detection in health care	Any treatment	Hospital treatment	Medication	Psychotherapy	Treatment at eating disorder specialist unit
Specified eating or feeding disorder								
Anorexia nervosa^a^	Females	44	24	22	6	7	6	3
	Males	2	2	2	1	1	1	1
Bulimia nervosa^a^	Females	17	8	8	1	4	1	1
	Males	1	1	1	1	1	0	1
Binge eating disorder	Females	4	1	1	0	0	0	0
	Males	2	1	0	0	0	0	0
Other specified feeding or eating disorder								
OSFED atypical AN	Females	15	7	6	0	1	1	1
	Males	1	0	0	0	0	0	0
OSFED low frequence and/or limited duration BN	Females	3	0	0	0	0	0	0
	Males	0	0	0	0	0	0	0
OSFED low frequence and/or limited duration BED	Females	5	1	1	0	0	1	0
	Males	0	0	0	0	0	0	0
OSFED purging disorder	Females	9	1	1	0	0	0	0
	Males	0	0	0	0	0	0	0
Total OSFED	Females	32	9	8	0	1	2	1
	Males	1	0	0	0	0	0	0
Unspecified feeding or eating disorder								
	Females	32	1	1	0	0	1	0
	males	10	1	0	0	0	0	0
Any eating disord								
	Females	127	41	38	7	11	9	5
	Males	15	4	2	1	1	1	1

Abbreviations: AN, anorexia nervosa; BED, binge eating disorder; BN, bulimia nervosa; OSFED, other specified feeding or eating disorder; UFED unspecified feeding or eating disorder.

a Three individuals had both AN and BN diagnoses (two females, one male).

**TABLE 2:** The mean duration of eating disorders among all those with an eating disorder and those recovered and 5-year recovery rates among all those with an eating disorder, those who received treatment, and those who remained untreated

Eating disorder diagnosis	Mean duration of eating disorder (years), full sample (SD, range)	Mean duration of eating disorder of those recovered (years), (SD, range)	5-year recovery rate, full sample (95% CI)	5-year recovery rate for those treated for an eating disorder (95% CI)	5-year recovery rate for those not treated for an eating disorder (95% CI)	*p*-value for difference in 5year recovery rates for treated vs. untreated, (log-rank)
Anorexia nervosa	4.6 (3.4, 0.5–13)	2.0 (1.4, 0.5–6)	41.5% (28.2–58.0%)	40.0% (23.2–62.8%)	43.2% (24.4–68.1%)	*p* = 0.99
Bulimia nervosa	4.2 (2.9, 0.5–9)	1.3 (0.6, 1–2)	23.1% (8.1–55.8%)	28.6% (8.0–74.2%)	16.7% (2.5–72.7%)	*p* = 0.52
Binge eating disorder (BED)	4.0 (2.4, 1–7)	2.0 (1.4, 1–3)	40.0% (11.8–87.4%)	0%	57.0% (18.1–97.1%)	*p* = 0.47
Other specified feeding or eating disorder (OSFED)	3.9 (2.6, 0.5–11)	3.0 (2.1, 0.5–7)	43.1% (26.7–64.2%)	53.3% (24.4–87.5%)	38.9% (20.8–64.7%)	*p* = 0.33
Unspecified feeding or eating disorder (UFED)	3.2 (2.5, 0.5–9)	3.3 (3.1, 0.5–9)	42.6% (27.4–61.8%)	0%	43.3% (28.0–62.7%)	*p* = 0.54
Any eating disorder	4.0 (2.9. 0.5–13)	2.6 (2.3, 0.5–9)	40.7% (32.3–50.0%)	41.1% (27.2–58.7%)	40.5% (30.5–52.3%)	*p* = 0.66

*Note:* Females and males were analysed together.

Abbreviations: CI, confidence interval; SD, standard deviation.

## Key points

- Although eating disorders affected one in six females and one in 40 males in the community, only one-third of these cases were detected by healthcare providers, and even fewer received any treatment. Atypical disorders remain rarely recognized and treated.
- Eating disorder symptoms can be persistent for many sufferers; 5 years after disease onset, less than two-fifths of the females and two-thirds of the males had recovered.
- Real-world treatments available in healthcare may have limited effectiveness, as the likelihood of recovery within 5 years was 40 percent in both treated and untreated groups.

## Abbreviations:

AN: anorexia nervosa
Atypical AN: atypical anorexia nervosa
BED: binge eating disorder
BN: bulimia nervosa
CI: confidence interval
DSM-5: Diagnostic and Statistical Manual of Mental Disorders fifth edition
ED: eating disorder
EDNOS: eating disorder not otherwise specified
Mm-MAST: the Malmö-modified Michigan Alcoholism Screening Test
OSFED- BED: binge eating disorder of low frequency and/or limited duration
OSFED: other specified feeding or eating disorder
OSFED-BN: bulimia nervosa of low frequency and/or limited duration
PD: purging disorder
SCID: the Structured Clinical Interview for DSM-IV
SD: standard deviation
UFED: unspecified feeding or eating disorder
