# Supplementary material for: Detection, treatment, and course of eating disorders in Finland: A population-based study of adolescent and young adult females and males
Source: Eur Eat Disord Rev. Author manuscript; Available in PMC 2022 Sep 1. (PMC8349843; doi:10.1002/erv.2838)
Supplement: Supplementary Figure 2 [file NIHMS1707871-supplement-Supplementary_Figure_2.pdf]

Supplement 2. Recovery from eating disorders among those who received treatment for eating disorder versus those who remained untreated in each diagnostic group. Females and males were analyzed together.

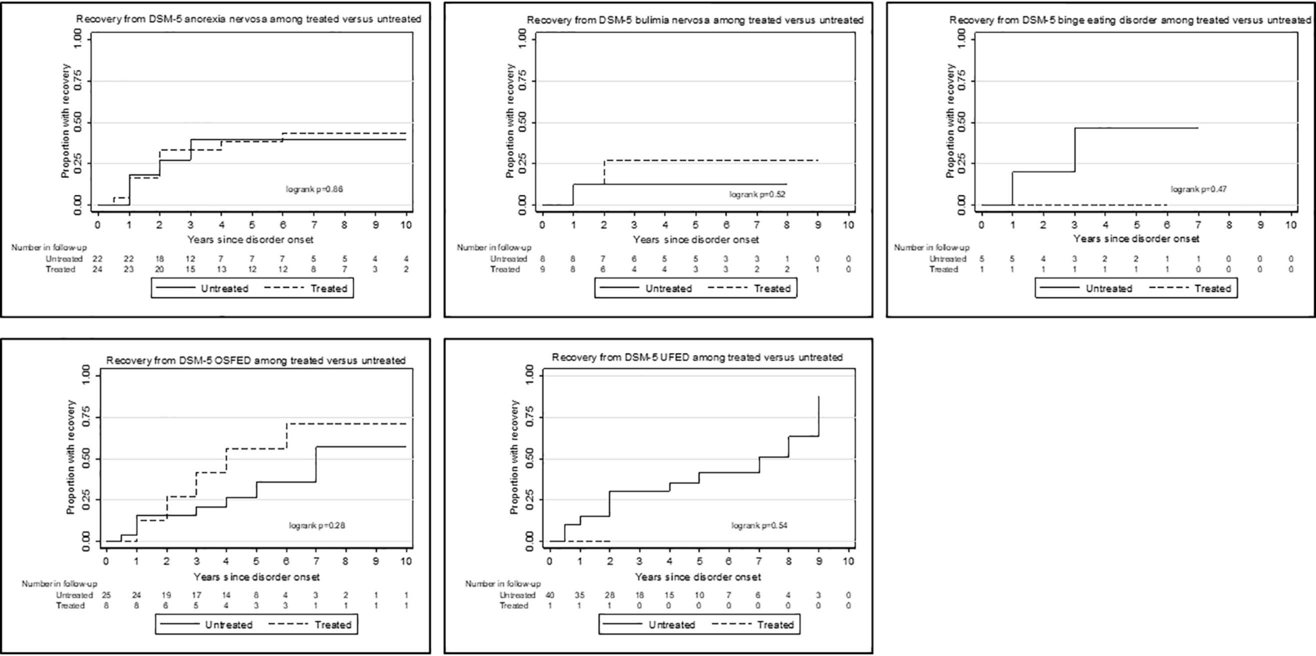

Abbreviations: OSFED, other specified feeding and eating disorders; UFED, unspecified feeding and eating disorders.
