# Supplementary material for: Detection, treatment, and course of eating disorders in Finland: A population-based study of adolescent and young adult females and males
Source: Eur Eat Disord Rev. Author manuscript; Available in PMC 2022 Sep 1. (PMC8349843; doi:10.1002/erv.2838)
Supplement: Supplementary Figure 1 [file NIHMS1707871-supplement-Supplementary_Figure_1.pdf]

Supplement 1. Recovery from other specified feeding and eating disorder (OSFED) subgroups. Females and males were analyzed together.

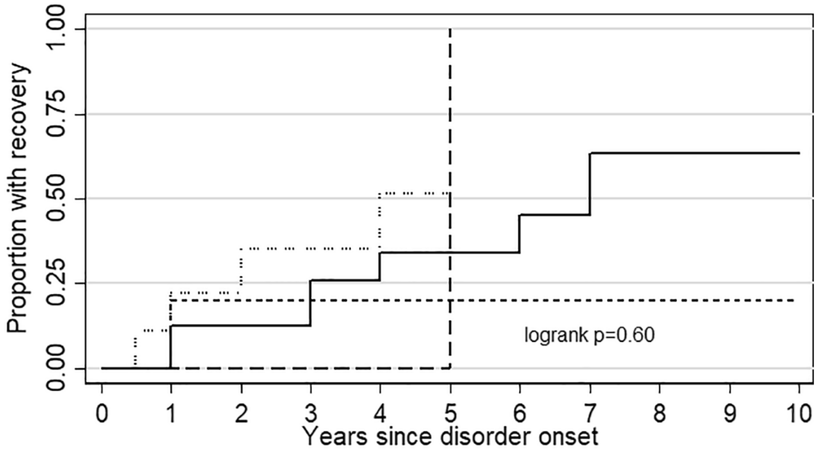

Number in follow-up

	0	1	2	3	4	5	6	7	8	9	10
Atypical AN	16	16	13	13	9	7	6	3	2	1	1
OSFED-BN	3	3	2	2	2	1	0	0	0	0	0
OSFED-BED	5	5	4	3	3	2	1	1	1	1	1
Purging Disorder	9	8	6	4	4	1	0	0	0	0	0

Abbreviations: Atypical AN, Atypical Anorexia Nervosa; OSFED-BN, Bulimia Nervosa of low frequency and/or limited duration; OSFED- BED, Binge Eating Disorder of low frequency and/or limited duration At the moment.
